# Global, regional, and national prevalence of hepatitis B infection in the general and key populations living with HIV: a systematic review and meta-analysis protocol

**DOI:** 10.1186/s13643-018-0815-5

**Published:** 2018-09-27

**Authors:** Steve Leumi, Jean Joel Bigna, Marie A. Amougou, Leopold N. Aminde, Ulrich Flore Nyaga, Jean Jacques Noubiap

**Affiliations:** 1 The Biotechnology Centre, University of Yaoundé I, Yaoundé, Cameroon; 2 School of Public Health, Faculty of Medicine, University of Paris Sud XI, Le Kremlin Bicêtre, Paris, France; 3 Department of Virology, Centre Pasteur of Cameroon, Yaoundé, Cameroon; 4 School of Public Health, Faculty of Medicine, University of Queensland, Brisbane, Australia; 5 Department of Internal Medicine and Specialties, University of Yaoundé I, Yaoundé, Cameroon; 6 Department of Medicine, University of Cape Town and Groote Schuur Hospital, Cape Town, South Africa

**Keywords:** Hepatitis B, HIV, AIDS, Key population

## Abstract

**Background:**

Hepatitis B causes death mainly due to liver disease worldwide. Human immunodeficiency virus increases the pathological effect of hepatitis viruses and potentiates re-activation of latent hepatitis infections as a result of reduced immunity. Because of the same modes of transmission shared by the two infections, HBV represents an important cause of co-morbidity and mortality among people living with HIV; hence, the aim of this review is to determine the prevalence of HBV among people living with HIV.

**Methods:**

This systematic review and meta-analysis will include cross-sectional, case-control, and cohort studies of patients positive for HBV and HIV irrespective of their countries. All pertinent articles published on hepatitis B in people living with HIV from January 1, 1990, to July 31, 2017, without any language restriction will be searched in PubMed/MEDLINE, Global Index Medicus Web of Science, and Excerpta Medica Database. Two review authors will independently assess the relevance of all titles and abstracts identified from the electronic searches. The study-specific estimates will be pooled through a random-effects meta-analysis model to obtain an overall summary estimate of the prevalence of HBV across studies. We will assess statistical heterogeneity and pool clinically homogeneous studies. On the other hand, we will evaluate statistical heterogeneity by the chi-squared test on Cochrane’s Q statistic. Symmetry of funnel plots and Egger’s test will be used to detect the presence of publication and selective reporting bias. In the case of publication bias, we will report estimates after adjustment on publication bias using the trim-and-fill method. We will assess inter-rater agreement between investigators for study inclusion, data extraction, and methodological quality assessment using Kappa Cohen’s coefficient. This protocol will comply with the guidelines for meta-analyses and systematic reviews of Preferred Reporting Items for Systematic Review and Meta-Analysis (PRISMA).

**Discussion:**

To our knowledge, this is the first systematic review and meta-analysis protocol to report the prevalence of HBV in people living with HIV. We believe its outcomes will be of utility in providing insights on the characteristics of HBV epidemic in people living with HIV, and draw more attention of public health services to this association.

**Systematic review registration:**

PROSPERO CRD42017073124

**Electronic supplementary material:**

The online version of this article (10.1186/s13643-018-0815-5) contains supplementary material, which is available to authorized users.

## Background

The human immunodeficiency virus (HIV) targets the immune system and weakens people’s defense systems against infections. Infected people gradually become immunodeficient since the virus destroys and impairs the function of immune cells which is measured by CD4 cell count. The most advanced stage of the infection is acquired immunodeficiency syndrome (AIDS) and is defined by the development of certain cancers, infections, or other severe clinical manifestations. HIV/AIDS pandemic represents a global public health concern as this has already led to the death of more than 35 million people thus far and millions of new cases are reported each year [1]. In 2015, 45% of HIV new infections was found in key populations which includes sex workers, people who inject drugs, transgender people, prisoners, and gay men and other men who have sex with men, and their sexual partners [2]. Analysis of the data available to the United Nations programme on HIV/AIDS suggests that more than 90% of the global HIV new infections in 2014 were among members from this group which has been marginalized in society for years [2].

HIV infection can accelerate the course of concurrent sexually transmissible infections like hepatitis B; this results in a faster progression to fibrosis and cirrhosis [3] and makes liver disease one of the most important non-AIDS causes of death in people living with HIV [4]. Since HIV and HBV have similar transmission routes, co-infections are common and are associated with long-term morbidity and mortality that exceeds the impact of infections with either one of the viruses alone [5].

Even though HBV and HIV are more prevalent in key populations than in general population around the world, there are regions like Southern Africa where this prevalence is particularly high and data available about the prevalence of HBV among people living with HIV is very limited [1], whereas addressing this issue requires an in-depth inventory of the current state of this co-infection which would help improve the management and prevention strategy. In this context, we decided to present a systematic review and meta-analysis protocol on the prevalence of hepatitis B among people living with HIV.

## Methods and design

Centre for Reviews and Dissemination recommendations will be used as guidelines to conduct this review [6]. The guidelines for meta-analyses and systematic reviews of Preferred Reporting Items for Systematic Review and Meta-Analysis (PRISMA) will serve as the template for reporting the present review [7]. For the present protocol, the PRISMA for Protocol was used for the reporting [8]. An additional file shows the PRISMA for protocol checklist (see Additional file 1). This review protocol is registered in the PROSPERO International Prospective Register of systematic reviews, registration number CRD42017073124. To avoid the possibility of outcome reporting bias, we do not intend to make any amendments to the protocol. Any amendments during review process will be reported transparently.

### Eligibility criteria

#### Types of studies

The types of studies are cross-sectional, case-control, and cohort studies.

#### Types of participants

The types of participants are people living with HIV.

#### Types of outcome measures

The types of outcome measures are studies reporting the percentage of people infected with HBV among people living with HIV or enough data (sample size and number of events—HBV infection cases) to compute this estimate.

#### Additional criteria

- Study period should be between January 1, 1990, and July 31, 2017.
- All languages will be considered.
- Duplicates: for studies published in more than one paper, the most comprehensive one with the largest sample size will be considered.
- Studies whose full key data (sample size and number of events) will not be accessible even after request from the authors will be excluded.

### Search methods for identification of studies

The search strategy will be implemented in two stages:

#### Electronic search

PubMed/MEDLINE, Global Index Medicus Web of Science, and Excerpta Medica Database will be searched to identify all relevant articles published on hepatitis B among people living with HIV from January 1, 1990, to July 31, 2017, without any language restriction. A search strategy based on the combination of relevant terms will be applied. Medical subject headings and other search words or their variants will include the following: “hepatitis B”, “HIV”, and “AIDS”.

#### Searching other sources

References lists of studies that will be included and relevant reviews will be manually searched to identify additional studies. Study authors will be contacted for further information on their studies and to enquire whether they are aware of any other published or ongoing studies.

#### Selection of studies for inclusion in the review

The results of the electronic search will be aggregated in a reference list in Endnote X7. The reference list will then be exported to the Rayyan systematic review web-based application (http://rayyan.qcri.org) followed by the removing of duplicates. Two review investigators will independently assess the relevance of all titles and abstracts identified from the electronic searches. Full text copies of all articles judged to be potentially relevant from the titles and abstracts will be retrieved. Two review investigators will independently assess these articles for inclusion. Any disagreements will be resolved by discussion between the two review investigators or by arbitration of a third review investigator. The reference lists of key publications will be also reviewed. Where they will be a doubt on the eligibility of a study, authors will be contacted for further details. For studies in other languages than English and French, we will use Google Translate for translation.

#### Data extraction and management

Two investigators will extract data pertaining to

- Author details: name of first author and publication year.
- Study characteristics: country, study design, setting, data source, sampling method, data collection period, response rate, timing of data collection, and diagnostic method for HBV. World Health Organization (WHO) region and level of income will be assigned depending on countries of recruitment.
- Participants’ characteristics: inclusion criteria, mean and/or median age, age range, proportion of male, specific profile of the study population including pregnant women, injecting drug users, male who have sex with male, transgender, female/male sex workers, and prisoners.
- HIV-related data: mean and/or median time since diagnosis of HIV infection, mean and/median CD4 cell count, and proportion of patients on ART.
- HBV characteristics: diagnostic method used, prevalence, number of participants tested, and diagnosed with HBV.

Where only primary data (sample size and number of outcomes) will be provided, these data will be used to calculate the prevalence. Data will be extracted using a preconceived, piloted, and standardized data abstraction form. Disagreements between review investigators will be reconciled through discussion and consensus, or arbitration by a third review investigator whenever necessary. In case of multinational studies, the results will be separated to show the estimate within individual countries. When it will not be possible to disaggregate the data by country, the study will be presented as one and the countries in which the study was done will be shown.

### Appraisal of the quality of included studies

The methodological quality of included studies will be evaluated using an adapted version of the risk of bias tool for prevalence studies developed by Hoy and colleagues [9]. A score of 0–3, 4–7, and 8–10 rated the risk of bias as high, moderate, and low (see Additional file 2). Two review investigators will independently assess study quality, with disagreements resolved by consensus or arbitration of a third review investigator.

### Data synthesis including assessment of heterogeneity

Data will be analyzed using Stata software (Stata Corp V.13, Texas, USA). Unadjusted prevalence and standard errors will be recalculated based on the information of crude numerators and denominators provided by individual studies. To keep the effect of studies with extremely small or extremely large prevalence estimates on the overall estimate to a minimum, the variance of the study-specific prevalence will be stabilized with the Freeman-Tukey single arc-sine transformation before pooling the data with the random-effects meta-analysis model [10]. Heterogeneity will be evaluated by the chi-squared test on Cochrane’s Q statistic [11], which will be quantified by *I*-squared values, assuming that *I*-squared values of 25%, 50%, and 75% being representative of low, medium and high heterogeneity respectively [12]. When substantial heterogeneity will be detected, subgroup analysis and meta-regression will be performed to investigate the possible sources of heterogeneity using the following variables: age, sex, study design, sampling method, data collection period, timing of data collection, diagnostic method for HBV, study setting, WHO region, level of income, ART regimens, and study methodological quality. Symmetry of funnel plots and Egger’s test will be done to assess the presence of publication and selective reporting bias [13]. A *p* value < 0.10 will be considered indicative of statistically significant publication bias. In the case of publication bias, we will report estimates after adjustment on publication bias using the trim-and-fill method [14]. We will assess inter-rater agreement between investigators for study inclusion, data extraction, and methodological quality assessment using Kappa Cohen’s coefficient [15].

## Discussion

To the best of our knowledge, this will be the first systematic review and meta-analysis to report the prevalence of Hepatitis B in people living with HIV with emphasis on key populations. The findings of this study can help public health stakeholders develop more efficient strategies to curb the burden of this co-epidemic.

One of the limitations of this work is the fact that some of those initially HBV infected can spontaneously clear the infection and will be diagnosed as negative except if molecular diagnostic techniques are used. This is the case in many low- and middle-income countries where the diagnosis rate is low, and the majority of those infected remain undiagnosed until they develop serious liver problems [16]. Another limitation is the roll-out of the prophylactic HBV vaccination that has been variably introduced in many regions of the world over the past two decades; HBV prevalence will doubtless continue to change in accordance with the success of this vaccine campaign [17]. Given the limited resources available for population screening efforts, epidemiological data should guide who should be prioritized for testing and we hope the current review will contribute in this regard.

## Additional files

Additional file 1: Preferred Reporting Items for Systematic Review and Meta-Analysis for protocol checklist. (DOCX 32 kb)

Additional file 2: Newcastle-Ottawa quality assessment scale. (DOCX 14 kb)

## Abbreviations

AIDS: Acquired immune deficiency syndrome
ART: Antiretroviral therapy
HBV: Hepatitis B virus
HIV: Human immunodeficiency virus
WHO: World Health Organization
